# Wavefront‐sensorless adaptive optics with a laser‐free spinning disk confocal microscope

**DOI:** 10.1111/jmi.12976

**Published:** 2020-11-20

**Authors:** Syed Asad Hussain, Toshiki Kubo, Nicholas Hall, Dalia Gala, Karen Hampson, Richard Parton, Mick A. Phillips, Matthew Wincott, Katsumasa Fujita, Ilan Davis, Ian Dobbie, Martin J. Booth

**Affiliations:** ^1^ Department of Engineering Science University of Oxford Oxford UK; ^2^ Department of Applied Physics Osaka University Osaka Japan; ^3^ Micron Advanced Bioimaging Unit, Department of Biochemistry University of Oxford Oxford UK; ^4^ Department of Biochemistry University of Oxford Oxford UK

**Keywords:** Wavefront‐sensorless, Adaptive Optics, Confocal Microscope, Deformable Mirror, Laser‐Free Spinning Disk Microscope

## Abstract

Adaptive optics is being applied widely to a range of microscopies in order to improve imaging quality in the presence of specimen‐induced aberrations. We present here the first implementation of wavefront‐sensorless adaptive optics for a laser‐free, aperture correlation, spinning disk microscope. This widefield method provides confocal‐like optical sectioning through use of a patterned disk in the illumination and detection paths. Like other high‐resolution microscopes, its operation is compromised by aberrations due to refractive index mismatch and variations within the specimen. Correction of such aberrations shows improved signal level, contrast and resolution.

## 1 INTRODUCTION

Three‐dimensionally resolved fluorescence imaging is a vitally important method in biomedical microscopy. Several methods of optically sectioning widefield microscopes are available as complementary approaches to the widely used laser point‐scanning confocal microscopes. Many of these methods are, in essence, parallelised versions of the confocal laser scanning microscope, based upon the spinning Nipkow‐Petran disk^1^ ^,^ ^2^. Another approach uses lamp or light emitting diode (LED)‐based illumination in conjunction with a patterned disk placed in the illumination and imaging paths;^3^, ^4^, ^5^ this optical sectioning approach relies upon correlations between the patterns used in the illumination and detection paths. All of these methods suffer from problems caused by system and specimen‐induced aberrations, which arise from the inhomogeneous refractive index structure of thick specimens. As in all microscopes, such aberrations affect imaging quality, through loss of resolution and contrast.

Adaptive Optics (AO) has been introduced into microscopes to correct aberrations and restore image quality.^6^, ^7^, ^8^, ^9^ An adaptive element, such as a deformable mirror or liquid crystal spatial light modulator, is built into the microscope in order to compensate the aberrations introduced by the specimen.^6^ Different AO correction methods have been implemented for different microscope modalities. A common approach is to use the so‐called wavefront‐sensorless AO methods (or ‘sensorless AO’ for short) in microscopes.^9^ These methods estimate the best correction for the microscope by inferring the input aberrations from a sequence of images taken when a sequence of predetermined aberrations is applied with the adaptive element. The optimal design of such AO methods depends strongly on the nature of aberrations and the image formation process of the microscope.

This form of sensorless AO has been implemented previously in a Nipkow‐Petran type widefield microscope,^10^, ^11^, ^12^ which has imaging properties closely related to the confocal microscope. It has also been demonstrated in widefield and superresolution structured illumination microscopes.^9^ However, such aberration correction has not yet been shown in the correlation disk type microscopes, which have considerably different image formation processes. In this paper, we demonstrate a practical implementation of AO in this microscope, covering both hardware and control aspects, by incorporating a custom deformable mirror‐based AO unit with a Clarity microscope module (Aurox Ltd, UK).

### 1.1 Imaging process and effects of aberrations

**FIGURE 1:**
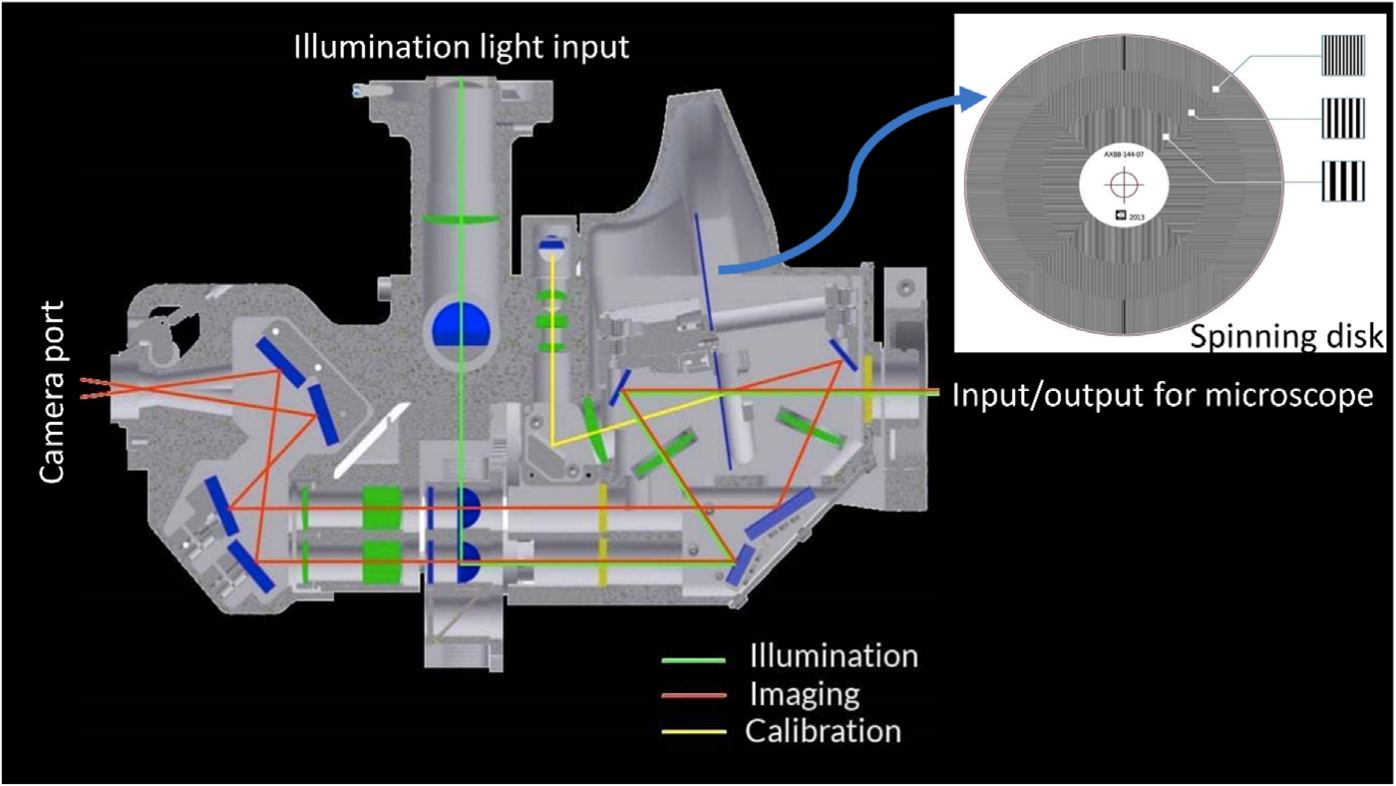
Cross sectional view of the Aurox clarity module, which is placed between the microscope port and the camera and light source. The spinning disk inside the system includes three patterns with different spacings for different sectioning strengths. (Modified with permission from Aurox Ltd., Copyright Aurox Ltd.)

The correlation disk microscope is fundamentally a widefield fluorescence incoherent imaging system. A disk is placed in an image plane in the common illumination and imaging path (Figure 1). The disk is imprinted with a binary pattern that either reflects or transmits the incident light. In the illumination path, the light generated by the LED source impinges upon the disk; part of this light is reflected by the pattern and discarded; the other part passes through the pattern and illuminates the specimen. The patterned illumination selectively excites fluorescence in the focal plane of the specimen. It is important to note that this pattern only appears near the focal plane and in out‐of‐focus planes the illumination is uniform. The fluorescence emitted by the specimen is imaged back onto the disk. The light that originated from the focal plane passed through the pattern on the disk. The remaining emission is reflected by the disk into a separate beam path. The paths are arranged such that both images (transmitted through and reflected off the disk pattern, respectively) fall onto two halves of the camera chip. The two images are aligned with the help of calibration pattern inside the box and shown in yellow in Figure 1, according to the manufacturers’ standard procedures. The disk is rotated at a speed much faster than the camera frame rate in order to average out the appearance of the pattern on the camera images.

It has been shown^3^ ^,^ ^4^ that the disk‐transmitted image $I_{T}$ is in effect a sum of a conventional microscope image $I_{conv}$ and a sectioned image $I_{sect}$, whereas the disk‐reflected image $I_{R}$ is the conventional image minus the sectioned image:

(1) $$\;I_{T}=\;I_{conv}+I_{sect},$$

(2) $$\;I_{R}=\;I_{conv}-I_{sect}.$$

Following appropriate image registration processes, it is thus possible to extract the conventional and sectioned images simultaneously as

(3) $$\;I_{conv}=\;0.5\left(I_{T}+I_{R}\right)\;,$$

(4) $$\;I_{sect}=\;0.5\left(I_{T}-I_{R}\right)\;.$$

The exact nature of the sectioned image depends upon the properties of the microscope optics, including objective lens numerical aperture (NA), and of the disk pattern. The Aurox Clarity disk patterns consist of parallel, equally spaced stripes with unit mark‐space ratio. Different patterns are available on the disk with different stripe spacings, which provide different sectioning ratios, with finer patterns providing stronger sectioning, albeit at lower contrast. This parallels closely the phenomena seen when varying the pinhole size in a point scanning confocal microscope, where a smaller pinhole provides better sectioning, albeit with lower signal level.

When aberrations are present in this microscope, they will affect both the illumination and the imaging paths. On illumination side, the aberrations will cause a blurring of the disk image in the specimen, thus resulting in lower contrast in the structured illumination pattern. On the detection side, the image of the fluorescence distribution onto the disk will be blurred, such that the distinction between the disk‐transmitted image and the disk‐reflected image becomes less clear. $I_{T}$ and $I_{R}$ become weaker, and the signal level (and hence signal to noise ratio) of $I_{sect}$ is reduced, coupled with compromised resolution – particularly in the axial direction. At the same time, the mean signal level in $I_{conv}$ is unaffected, although resolution and contrast are reduced. These phenomena mirror those seen in confocal and conventional microscopes, respectively, in the presence of aberrations.

### 1.2 Experimental system

**FIGURE 2:**
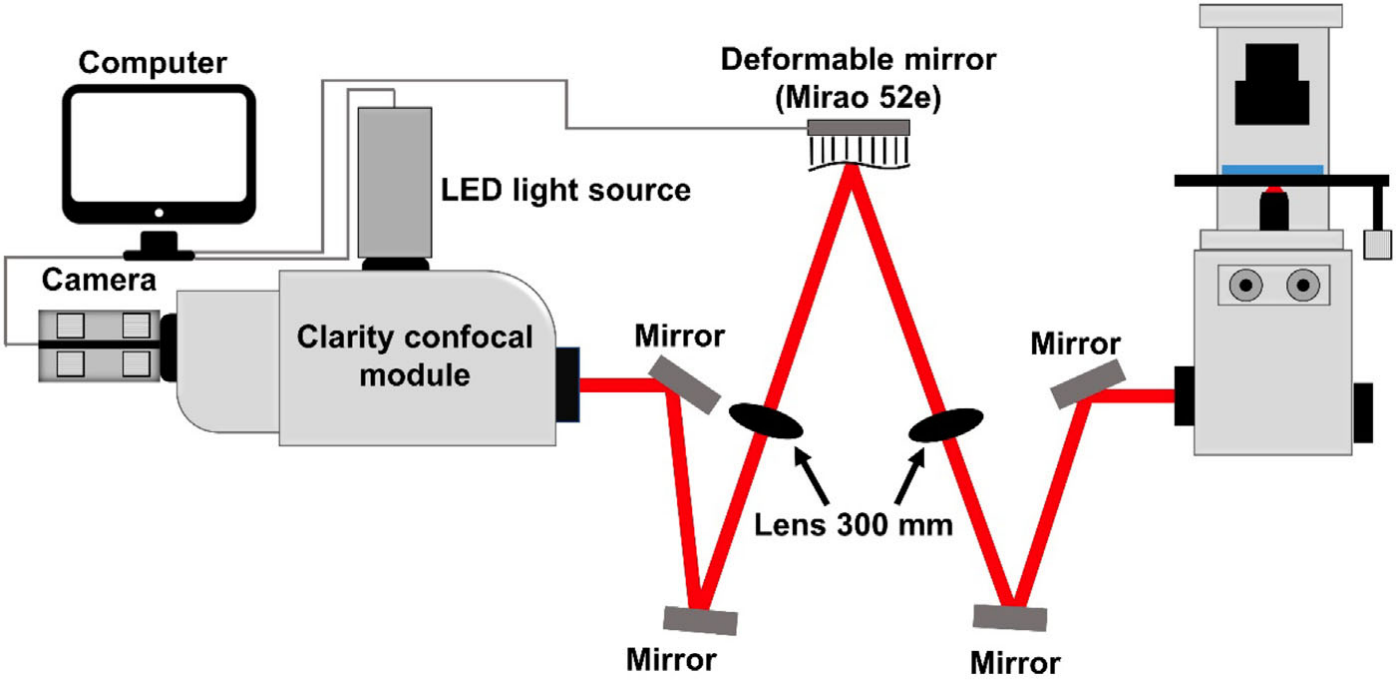
The experimental set‐up, consisting of a microscope base, an intermediate imaging system incorporating the deformable mirror, the confocal microscope module, a camera, a light source and a personal computer

In normal operation, the Clarity disk module would be attached directly to the microscope side port. In our case, we separated the module from the microscope base (iX71, Olympus, Japan) in order to slot in the AO system. To do this, we created an intermediate image plane into which we could insert the DM (Mirao 52e, Imagine Optics, France) (Figure 2). The additional optics were designed so that the DM was imaged on the objective pupil plane within the microscope. Additionally, the image plane near the camera port of the microscope was reimaged onto the input port of the Clarity module using a 4f lens system. The 4f system used two 300 mm focal length achromatic doublets (Thorlabs, USA) to form a unity magnification telescope. The DM was placed in the intermediate pupil plane of this 4f system. Imaging was performed using an oil immersion microscope objective (PlanApo, Olympus, Japan; 60× magnification, NA 1.4). The focussing depth could be controlled using a piezo z‐stage (P‐736.ZR1S PI‐nano Z Microscope Scanner, Physik Instrumente, Germany) attached to the setup. The stage was able to move with a step size of 0.2 nm. Images from both the transmitted and reflected emission light paths were captured by the camera (Prime BSI, Photometrics, UK), which has pixel size of 6.5 µm × 6.5 µm; the area of the whole sensor area was 176.89 mm^2^ (13.3 mm × 13.3 mm or 18.8 mm diagonal). As described earlier, the two images were directed to the sensor in such a way that the two images were detected separately on the two halves of the sensor. The sectioned (confocal) image was obtained using Equation (4). For the study presented here, we used a commercial LED source (PE 300 ultra, CoolLED, UK) to illuminate the sample. The desired excitation and emission wavelengths were selected by using set of dichroic filter cubes. In our case we used the following four dichroic filters cubes:

- Dichroic filter 1: Excitation 466 nm/FWHM 40 nm, Emission 525 nm/FWHM 45 nm
- Dichroic filter 2: Excitation 554 nm/FWHM 23 nm, Emission 609 nm/FWHM 54 nm
- Dichroic filter 3: Excitation 578 nm/FWHM 21 nm, Emission 641 nm/FWHM 75 nm
- Dichroic filter 4: Excitation 392 nm/FWHM 23 nm, Emission 447 nm/FWHM 60 nm

The deformable mirror was calibrated by using a technique based on deflectometry.^13^ ^,^ ^14^ The control matrix derived at the end of the protocol was used for control of the DM. This was obtained by generating Zernike polynomials. The whole experimental setup was controlled using a personal computer.

### 1.3 Adaptive optics strategy

Aberrations were corrected using the deformable mirror via a sensorless AO method, as has already been applied to other adaptive microscopes;^9^ ^,^ ^15^, ^16^, ^17^ https://aomicroscopy.org/). For each aberration mode being corrected, a small number of images are taken with a different amount of the chosen mode applied with the DM. For each image, an image quality metric was determined. These image quality metric values were used to fit a function – in the simplest case, a Gaussian – and the peak of the function is taken to be the optimal correction for the current mode. Different modes were then corrected in sequence.

**FIGURE 3:**
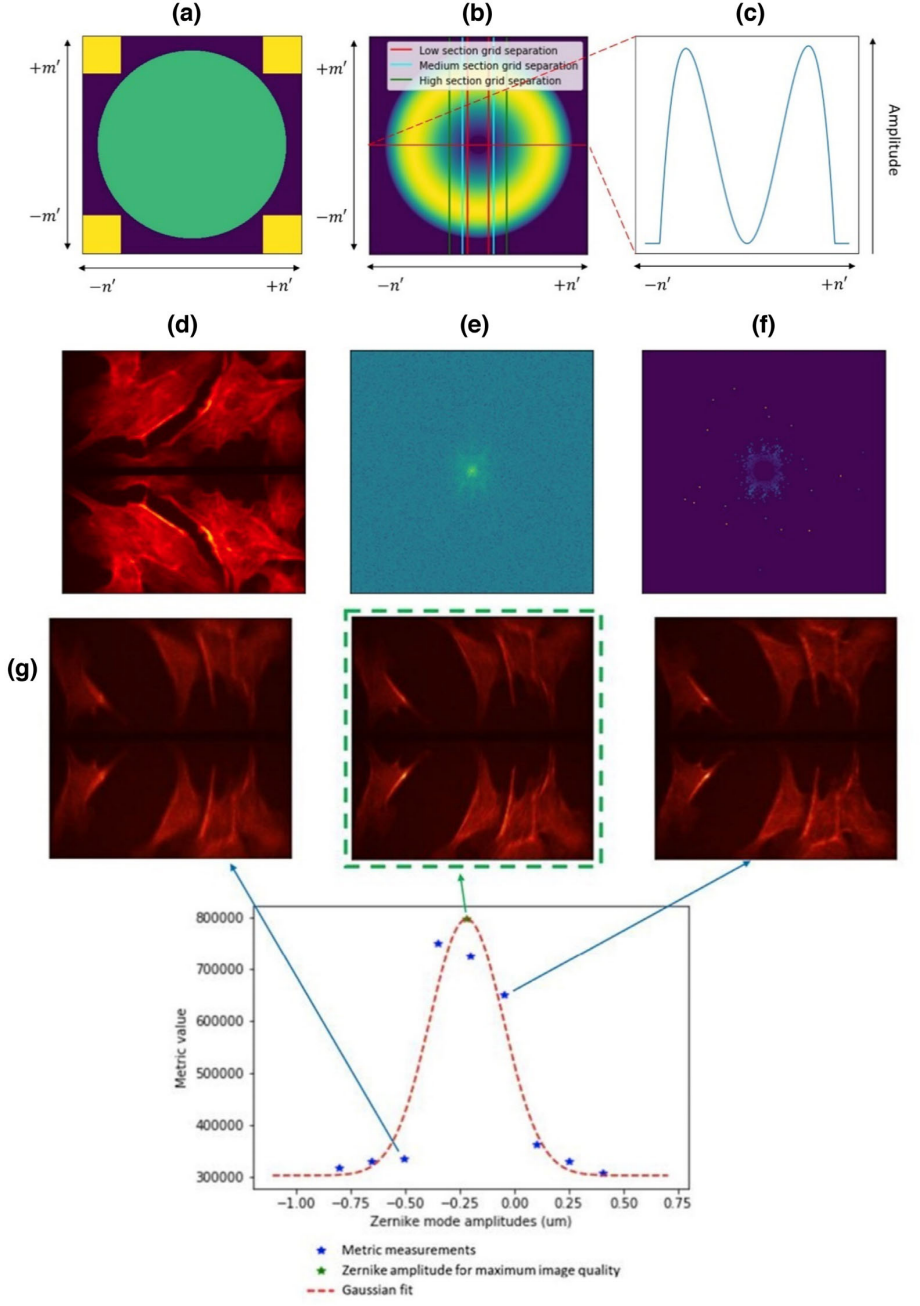
(a) $m^{\prime }$ and $n^{\prime }$ represent the spatial frequencies corresponding to the x and y axis respectively. Visualisation of the binary mask, $\mu_{n^{\prime },m^{\prime }}$, shown in green and the noise sampling regions for determining δ, shown in yellow. (b) Visualisation of the weighting mask, $\rho_{n^{\prime },m^{\prime }}$; also shown as positions on the horizontal axes are the spatial frequencies of the three patterns on the microscope disk. (c) Cross section of $\rho_{n^{\prime },m^{\prime }}$ showing that for calculation of the optimisation metric, the middle‐high spatial frequencies are amplified while the very high spatial frequencies (close to 1/*θ*) are suppressed. (d) An example of a raw image obtained by using bovine pulmonary artery endothelial (BPAE) cells. The image in D shows the raw image obtained by the camera; the upper half contains $I_{T}$ and the lower half $I_{R}.\;$(e) The power spectrum of D, $|{\hat{I}}_{n^{\prime },m^{\prime }}{|}^{2}$ where ${\hat{I}}_{n^{\prime },m^{\prime }}$ is the discrete Fourier transform of D. (f) The power spectrum with both $\tau_{n^{\prime },m^{\prime }}$ and $\rho_{n^{\prime },m^{\prime }}$ applied. This amplifies middle‐high spatial frequencies presented in E and thresholds out all spatial frequencies outside of the OTF radius. The image quality metric, S, is the sum of all pixels in F. (g) An example showing the variation of image quality metric versus the applied correction of horizontal coma (Zernike mode 3, OSA indexing). A Gaussian function was fitted to the data to estimate the position of the peak, corresponding to the optimal correction. The appropriate aberration correction was then applied to obtain the corrected image

This method was implemented in Microscope‐AOtools, an extension to the Python Microscope hardware control software.^19^ Microscope‐AOtools offers a suite of image quality metrics and optimisation algorithms. In this case, the image quality metric, *S*, was a weighted sum of the power of all spatial frequencies obtained from the image spectrum. *S* was defined as:

(5) $$S\;=\sum_{n^{\prime },m^{\prime }}\tau_{n^{\prime },m^{\prime }}\rho_{n^{\prime },m^{\prime }}{\left|{\hat{I}}_{n^{\prime },m^{\prime }}\right|}^{2},$$

where ${\hat{I}}_{n^{\prime },m^{\prime }}$ is the discrete Fourier transform of the image, $\;n^{\prime }=\;n-\frac{N-1}{2}$ and $m^{\prime }=\;m-\frac{M-1}{2}$ where *n* and *m* are the pixel indices ranging from 0 to *N* – 1 and *M* – 1, respectively. *N* and *M* are the number of pixels along each image dimension. $\tau_{n^{\prime },m^{\prime }}$ is the thresholding mask and is defined as

(6) $$\;\tau_{n^{\prime },m^{\prime }}=\left\{\begin{array}{c} 1,\;{\left|{\hat{I}}_{n^{\prime },m^{\prime }}\right|}^{2}>\delta \\ 0,\;{\left|{\hat{I}}_{n^{\prime },m^{\prime }}\right|}^{2}\leq \delta \end{array}\right)\;\;,$$

where *δ* is the noise floor threshold defined as

(7) $$\delta \;=\frac{\alpha }{P_{noise}}\;\sum_{l,k}{\left|{\hat{I}}_{l,k}\right|}^{2},$$

where $|{\hat{I}}_{l,k}{|}^{2}\;$is the power in the lth, kth spatial frequency where l and k are the coordinates of the pixels in the regions shown in yellow in Figure 3a. $P_{noise}$ is the total number of pixels in the yellow region and α is a user defined, arbitrary scaling factor of 1.125. The scaling factor was chosen to be certain we were only using spatial frequency content that was above the noise level.

$\rho_{n,m}$ is a weighting mask (Figure 3b) and is defined as

(8) $$\;\rho_{n^{\prime },m^{\prime }}=\sqrt{n^{\prime^{2}}+{m^{\prime }}^{2}}\;\left(1-e^{-\theta \gamma_{n^{\prime },m^{\prime }}}\right),$$

$$\;\gamma_{n^{\prime },m^{\prime }}=\mu_{n^{\prime },m^{\prime }}\;\left(\frac{1}{\theta }-\sqrt{{n^{\prime }}^{2}+{m^{\prime }}^{2}}\right),$$

$$\;\mu_{n^{\prime },m^{\prime }}=\left\{\begin{array}{c} 1,\;\sqrt{{n^{\prime }}^{2}+{m^{\prime }}^{2}}\leq \frac{1}{\theta }\\ 0,\;\sqrt{{n^{\prime }}^{2}+{m^{\prime }}^{2}}>\frac{1}{\theta } \end{array}\right)\;,$$

where $\mu_{n,m}$ is the binary mask shown in green in Figure 3a and *θ* is the resolution limit of the microscope given by $\theta \;=\frac{1.22\lambda }{2NA}\;$. The effect of *ρ* is to amplify the power in the middle‐high spatial frequencies of the optical transfer function (OTF) (shown in the Figure 3b). Figure 3c shows this by taking a cross section in the centre. This makes the metric sensitive to image details that are most affected by aberrations (ie structures with mid‐high spatial frequency content). The rationale for this choice of optimisation metric is based upon the image formation process of the microscope, which relies upon the high‐fidelity reproduction of the structured illumination pattern in the specimen and its imaging back onto the disk. The pattern frequencies are set to be in the mid‐range of spatial frequencies covered by the OTF support. The power in low frequencies is barely affected by aberrations and the highest frequencies tend to be dominated by noise. Hence, the enhancement of the mid‐range frequencies in the optimisation should provide appropriate feedback for aberration correction.

The metric was calculated using the entire image captured by the camera. Optimisation was performed using the raw image data (rather than the processed sectioned or conventional images), as all of the necessary information for optimisation is contained within the image spectrum of the raw data. Figure 3d shows an example of a raw image for which we measure the image quality metric. Figure 3e shows the power spectrum, $|{\hat{I}}_{n^{\prime },m^{\prime }}{|}^{2}$, of the image and Figure 3f shows the power spectrum with $\tau_{n^{\prime },m^{\prime }}$ and $\rho_{n^{\prime },m^{\prime }}$ applied.

As described above, to obtain the optimum correction for a specific mode, a number of images were obtained with different amplitudes in the range of [−0.49μm,0.49μm] RMS wavefront deformation applied. The image quality metric was calculated for each image. The optimum correction for the mode was estimated and applied to the DM (see Section 2.2). This process was repeated for each Zernike mode. Figure 3g shows one such example along with the correction applied and obtained pictures. Finally, all these corrections were performed on one channel by using dichroic filter 1 (excitation 466 nm). Aberrations arising from the refractive index structures in the specimen should be corrected for all wavelengths by the deformable mirror, so correction performed on one channel should be valid for all.

## 2 RESULTS

### 2.1 Aberration correction for different sectioning patterns

**FIGURE 4:**
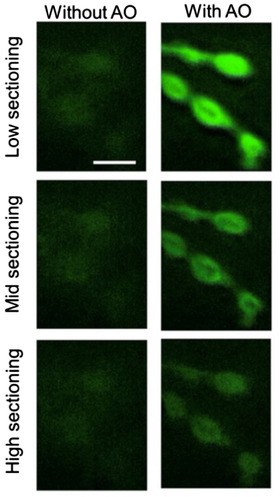
Experimental results when using different settings of the disk. The images were obtained from the NMJ sample. In this case we used only dichroic filter 1 (excitation 466 nm). Scale bar 10 µm

The microscope provides three disk patterns of differing spacing that can provide different optical sectioning. We demonstrate here the capabilities of the AO scheme in correcting each of these imaging modes. For all results in this paper, we implemented correction of the low order Zernike aberration modes OSA index 3, 5, 7, 8 and 12, which are oblique astigmatism, vertical astigmatism, vertical coma, horizontal coma and primary spherical, respectively. Comparisons with and without aberration correction were performed using same acquisition parameters. Figure 4 shows examples of such aberration correction using a *Drosophila* larva neuromuscular junction (NMJ) sample (see Section 2.2). In Figure 4, we see the synaptic boutons visualised with the Alexa488 fluorophore. In this case, we detected only one channel using dichroic filter 1 (excitation 466 nm).The panels on the left labelled ‘Without AO’ show the retrieved section image before aberration correction. Low sectioning using the coarsest disk pattern leads to a thicker optical section (2.5 µm, as specified by the manufacturer); the medium spaced pattern has a slightly smaller section (1.7 µm); the finest spacing corresponds to a thinner optical section (0.9 µm). In all of the ‘Without AO’ cases, the contrast and resolution are poor. This is due to the blurring out of the structured illumination and, hence, a reduction in the difference between the $I_{T}$ and the $I_{R}$ images.

On the other hand, after applying the AO correction we obtained the ‘With AO’ images that showed greater contrast and detail. The variation in brightness between the low, medium and high sectioning modes was due to there being less fluorescent marker within the narrower optical sections. The images were taken at the depth of 10 µm inside the sample. In each case, this clearly shows that the sensorless AO algorithm functions in all three modes. In the rest of this paper, we present only the results obtained by using the high sectioning mode of the microscope, which is expected to be more sensitive to aberrations.

### 2.2 Aberration correction varying with depth

**FIGURE 5:**
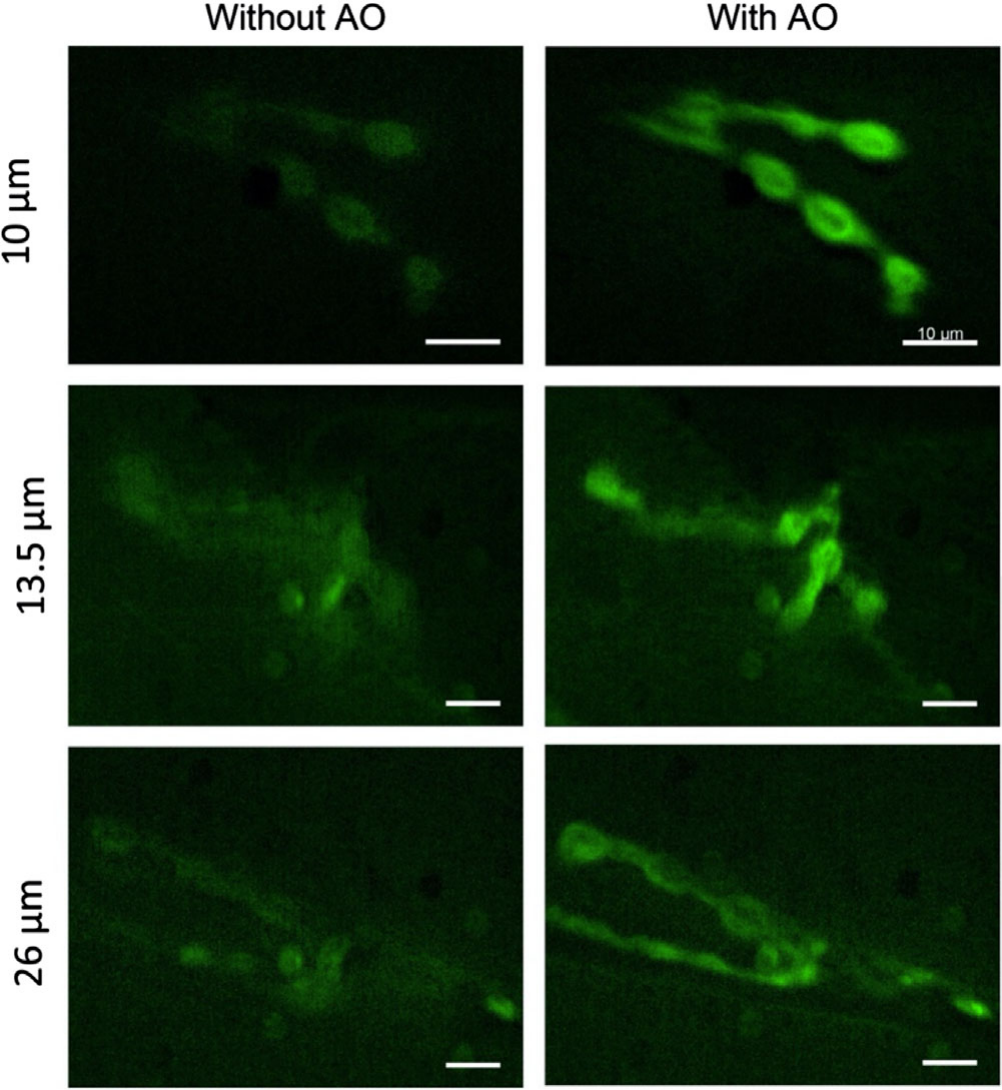
Images taken at depths of 10, 13.5 and 26 µm in the NMJ sample when dichroic filter 1 (excitation 466 nm) was used. Aberration correction improved imaging quality at all depths. Scale bars 10 µm

Figure 5 shows images of a *Drosophila* neuromuscular junction (NMJ; see Section 2.2 details) before and after correction at varying focussing depth. These images were taken at depths of 10, 13.5 and 26 µm inside the NMJ sample. In the figure, we see the synaptic boutons visualised with the Alexa488 fluorophore (see Section 2.2). One channel was acquired using dichroic filter 1 (excitation 466 nm).

At each depth in the sample, the AO correction provided improved contrast and resolution. When focussing to deeper planes into the sample, the signal level decreased even in the corrected images, which may be due to varying marker density, residual aberration or loss of signal through absorption or scattering as the light passes through more tissue.

**FIGURE 6:**
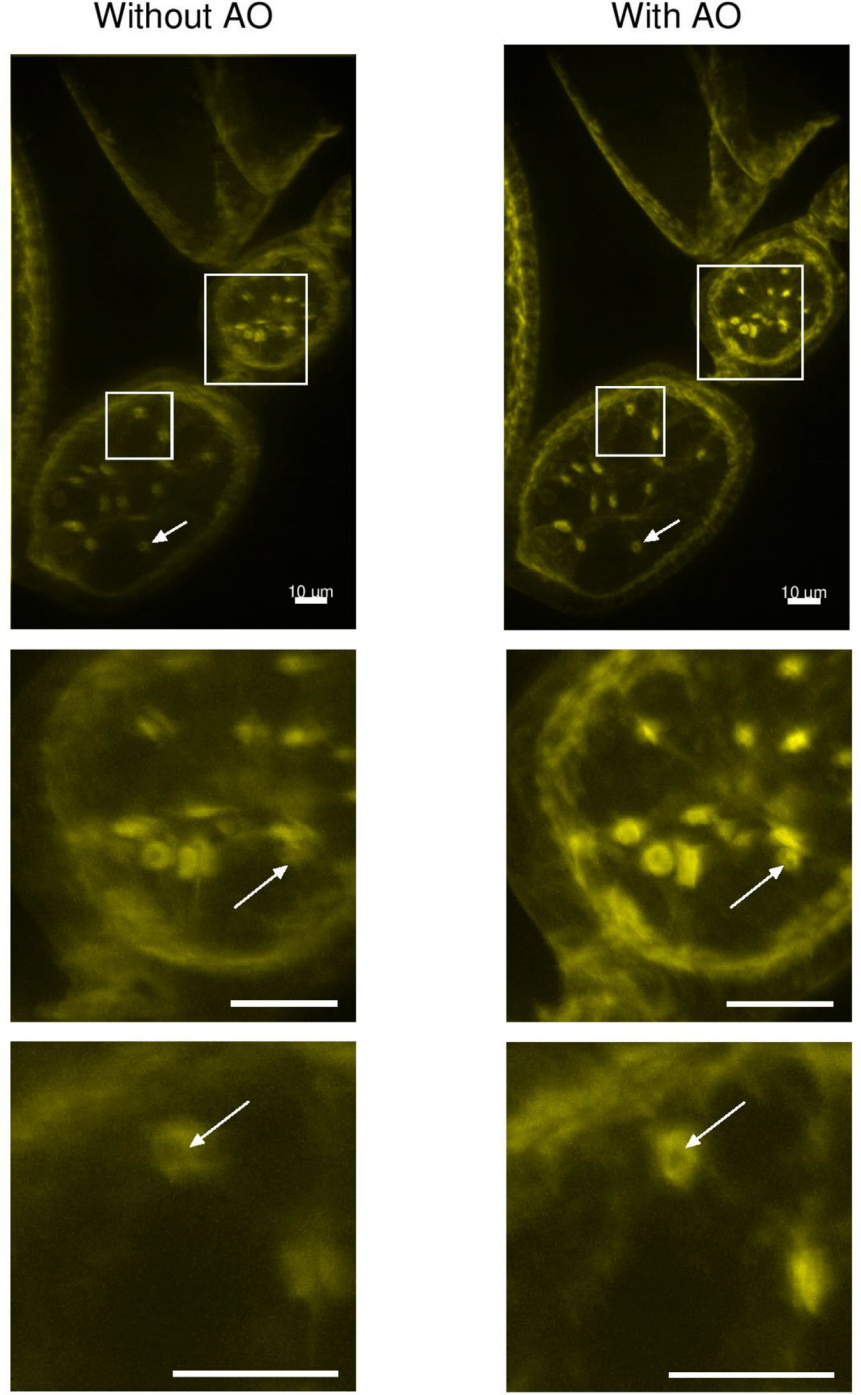
Egg chambers sample imaged without and with AO, first row (dichroic filter 1, excitation 466 nm). The middle and bottom represent zoom regions and we can see that small features are clearly visible when AO was applied. Scale bars 10 µm

Figure 6 shows the maximum intensity projections of 36 µm *z*‐stacks through a sample of early stage *Drosophila* egg chambers.^2‐5^ Correction was made at the centre and 180 *z* positions were taken. Ovaries were stained for actin in multiple colours using Phalloidin Alexa dye conjugates. Green emission was imaged using dichroic filter 1 (excitation 466 nm). Ovarioles were separated out to better visualise individual egg chambers. Arrows indicate actin labelling associated with ring‐canals, pores which connect adjacent nurse cells and the oocyte to adjacent nurse cells the egg chamber. As we can see in the zoomed version, the fine details that were not visible in the noncorrected version become visible in the corrected version. In this case, we would like to highlight the information in the lower panel in which the structure appears ring‐like after correction but looks filled in for the noncorrected image. Improvement in image quality for egg chambers is presented in Fig. 8.

**FIGURE 7:**
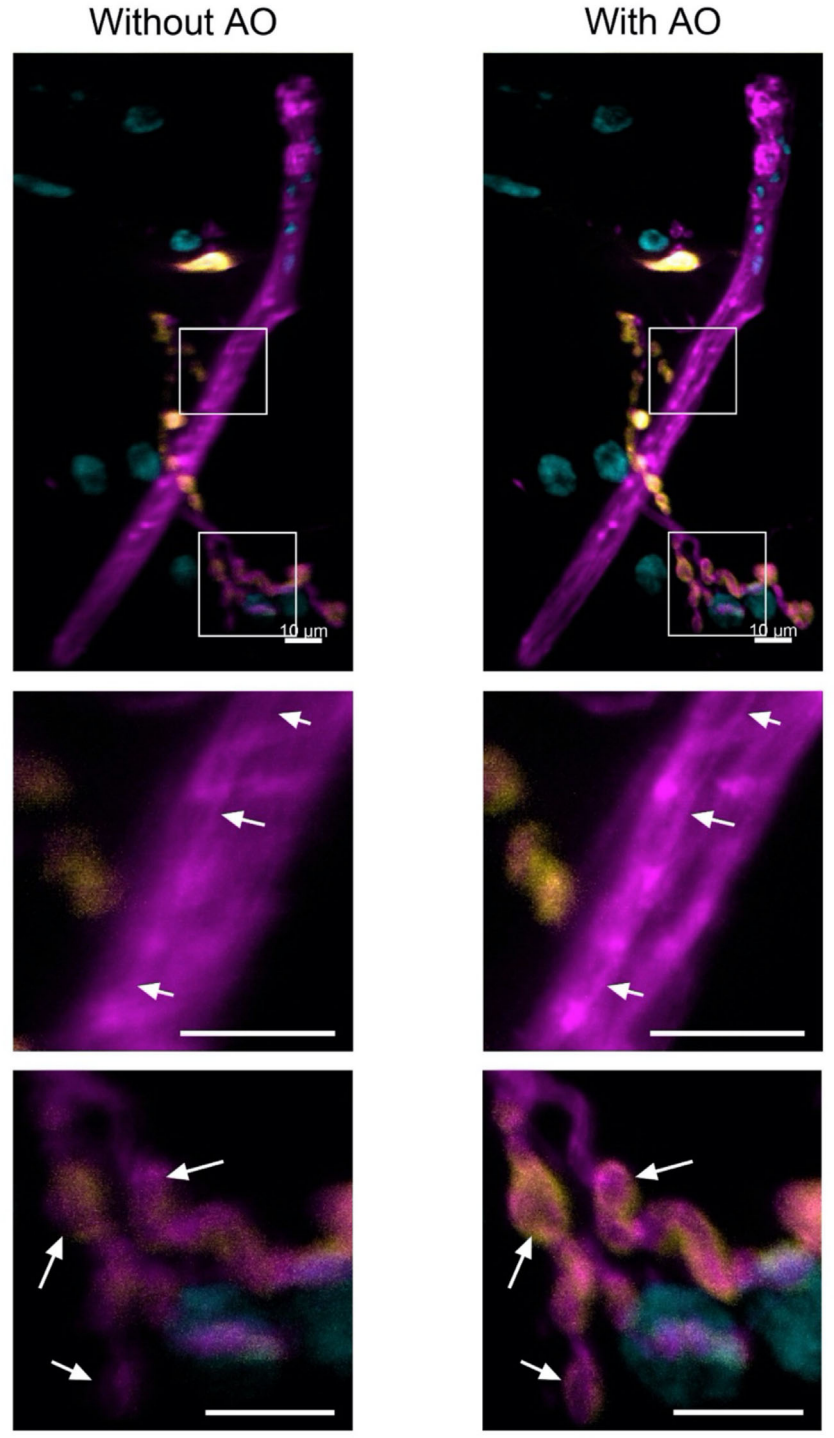
First row are maximum intensity projections of 20 µm *z*‐stacks through an NMJ sample. The middle row shows one region of interest in which we can see clear features indicated by arrows that were not visible without AO. The lower row shows a comparison of another region of interest in which individual round postsynaptic densities are clearly visible in the corrected images. Images were taken in three channels using dichroic filters 1, 2 and 4 (excitation 466, 554 and 392 nm, respectively); the NMJ synapse with the neuron is shown in magenta, postsynaptic density in yellow and the nuclei in cyan Scale bars 10 µm

Figure 7 shows results obtained for the maximum intensity projections of 20 µm *z*‐stacks through an NMJ sample. For this image, correction was performed at the centre of the stack and 200 *z* positions were taken for the stack. To gain more insight, we have presented zoomed images of sample regions. We can see in the middle panel that the fine details are only visible when we applied correction. In this case, we were able to see the structure of the fly nerve and synapse better. In the bottom right panel, we can see individual round postsynaptic densities – boutons – which are blurred and not well defined in the bottom left panel.

## 3 METHODS

**FIGURE 8:**
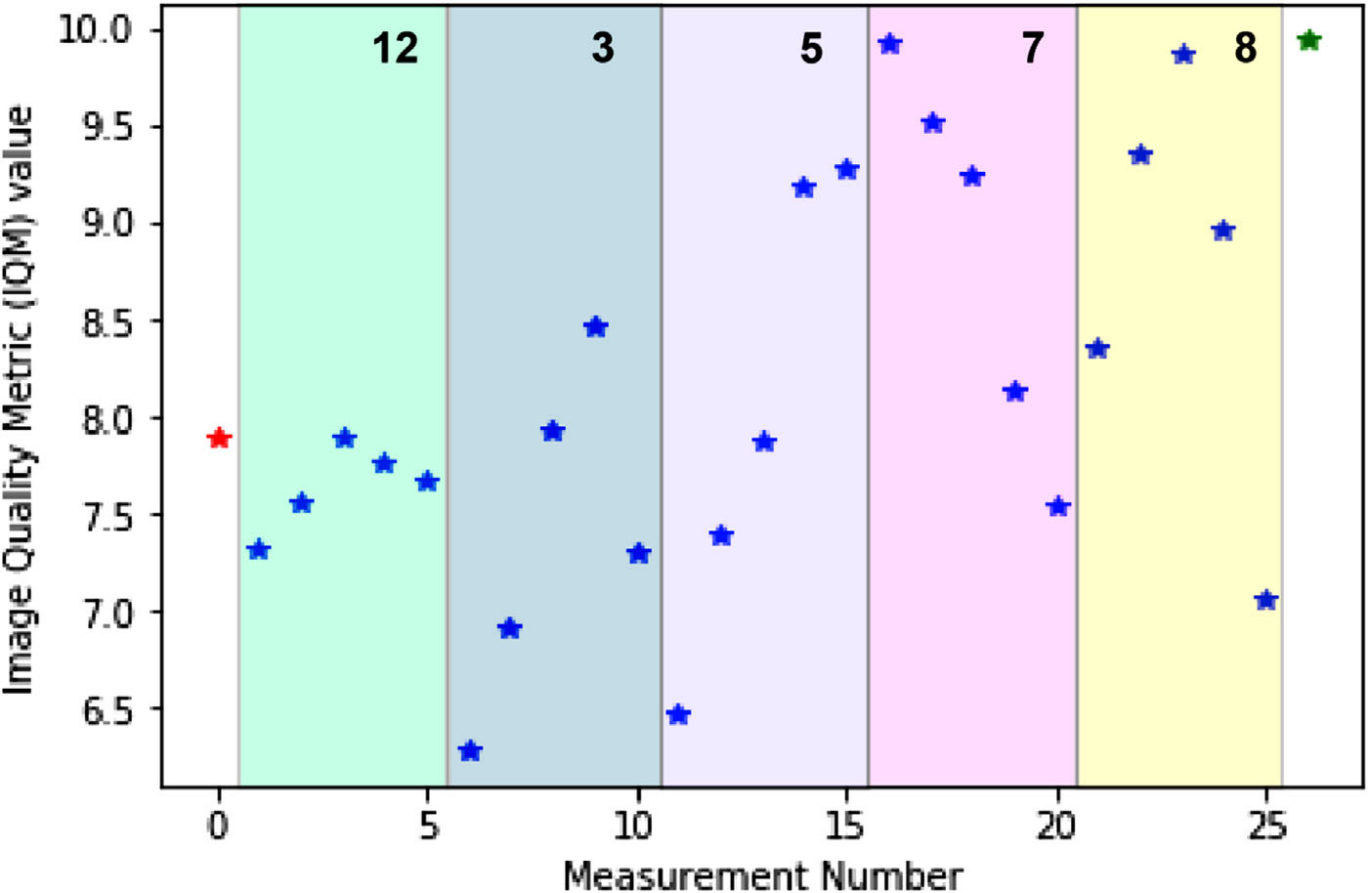
Image Quality Metric (IQM) obtained at each step of correction for a particular example using the egg chambers specimen. The shaded regions represent the sequence of correction for the different Zernike modes mentioned at the top of each region. The red star at extreme left shows the initial IQM and green star at extreme right shows the final IQM

For each round of correction, for a particular mode we took *N* measurements over the range [−0.49 µm, 0.49 µm]. We then fitted a Gaussian function using the SciPy python package,^20^ found the aberration coefficient peak value and applied the corresponding correction. We then progressed through the other modes, in a chosen order, Fig. 8. In all results in this paper, we implemented only one round of correction, as this provided noticeable improvements, although in some cases further rounds may also have been beneficial. One round of correction took approximately 11 seconds, although this time might be decreased with further optimisation. As it is possible that photobleaching could affect the correction estimate in this method, we also tested a scheme which used ascending and then descending coefficient values. This was found not to have a significant effect in realistic imaging scenarios.

Results were obtained from three samples: bovine pulmonary artery endothelial (BPAE) cells, *Drosophila* NMJ and *Drosophila* egg chambers. The detailed description of the samples is as follows:

**BPAE cells**: Cells (F36924, Invitrogen) were fixed and stained with variety of fluorescent dyes. Mitochondria were labelled with red‐fluorescent MitoTracker^®^ Red CMXRos (excitation 579 nm). Alexa Fluor^®^ 488 was used to stain F‐actin (excitation 505 nm). Finally, the nuclei were stained with blue‐fluorescent DAPI (excitation 358 nm).
**Egg chambers**: Ovaries were isolated from *Drosophila melanogaster* adult females straight into fixation medium and fixed in 4% PFA (prepared fresh from a stock solution of 16% paraformaldehyde, methanol free ultra‐pure EM grade, Polysciences) in Cytoskeleton Buffer^21^ ^,^ ^22^ for 10 minutes without added detergent.^23^ Ovaries were then heptane permeabilised and post fixed in 4% PFA in PBS for not more than 15 minutes, then washed three times with PBS/TX‐100, 0.05%. Actin was labelled with Phalloidin Alexa 488 (Phalloidin: stock 200 units/mL in Methanol, 6.6 µM) at between 5 and 20 µL stock in 200 µL for at least 45 minutes at RT (or O/N at 4°C). This was visible when dichroic filter 1 (excitation 466 nm) was used. Labelled ovarioles were washed three times with PBS TX‐100, 0.05%, and then finally with PBS before being preincubated at RT for at least 3 hours in 70% Vectashield. This was removed and replaced with fresh 70% Vectashield prior to samples being mounted under a coverslip raised on two thin strips of double‐sided sticky tape to avoid flattening.^24^ The edges of the coverslips were double varnished, and slides were stored at 4°C until use.
**NMJ**: The samples were prepared by following the protocol presented in Brent et al.^25^ Third‐instar *Drosophila melanogaster* larvae (Oregon‐R strain) were dissected in HL3 buffer with 0.3 mM Ca^2+^ to prepare a so‐called larval fillet. After this, larvae were fixed with paraformaldehyde and blocked using BSA. Larvae were stained overnight with HRP [conjugated to Alexa568 fluorophore, visible when dichroic filter 2 (excitation 554 nm) was used] to visualise the neurons, and primary mouse antibody against DLG – discs large – to visualise the postsynaptic density. The next day, the larvae were counterstained with secondary antibody to detect the DLG (donkey antimouse conjugated to Alexa488 fluorophore, visible when dichroic filter 1 (excitation 466 nm) was used), as well as DAPI to visualise the nuclei [visible when dichroic filter 4 (excitation 392 nm) was used]. The larvae were then washed and mounted in 65% vectashield (this dilution of vectashield is compatible with the oil immersion lens that was used).

## 4 CONCLUSION

We have introduced a sensorless AO correction scheme that is applicable to correlation disk microscopy. Aberration correction has been achieved by the optimisation of an image quality metric that is designed to be sensitive to spatial frequencies in the mid‐range of the OTF pass band, as these are the most important for effective imaging in this system. The scheme was shown to be suitable for different sectioning modes and fluorescence channels, as in all cases it was able to increase signal levels and reveal image details that were otherwise blurred. The AO methods should be widely applicable for other imaging applications, extending the capabilities of these microscopes for deep tissue imaging.

## FUNDING

This research was funded by the European Research Council (ERC) under the European Union's Horizon 2020 research and innovation programme (Grant agreement No. 695140, AdOMiS), Wellcome Trust (107457/Z/15/Z, 209412/Z/17/Z). Nicholas Hall is supported by funding from the Engineering and Physical Sciences Research Council (EPSRC) and Medical Research Council (MRC) (grant number EP/L016052/1). JST CREST program (Grant No. JPMJCR15N3). We also would like to thank Dr Phillipa Timmins from Aurox Ltd and Dr Jacopo Antonello from the University of Oxford for technical help.

## DISCLOSURES

Martin Booth declares a significant interest in Aurox Ltd.
